# Roles of Dementia and Vision Loss on Physical Resilience in Persons With Dementia After Hip Fracture

**DOI:** 10.1093/geroni/igaf122.1257

**Published:** 2025-12-31

**Authors:** Tingzhong (Michelle) Xue, Wei Pan, Cathleen Colón-Emeric, Michael Cary Jr., Eleanor McConnell

**Affiliations:** College of Nursing, UMass Amherst, Amherst, Massachusetts, United States; Duke University, Durham, North Carolina, United States; Duke University School of Medicine, Durham, North Carolina, United States; Duke University, Durham, North Carolina, United States; Duke University, Durham, North Carolina, United States

## Abstract

Older adults with dementia residing in nursing homes (NHs) are more likely to experience worse health outcomes after a hip fracture than those without dementia. How chronic stressors such as dementia and vision loss interact to influence physical resilience after acute injury is not well understood. Behavioral symptoms, common among NH residents with dementia, may worsen with the acute stressor of hip fracture, affecting rehabilitation and function recovery. However, the mechanisms of how vision loss and behavioral symptoms together affect physical function recovery after hip fracture are poorly understood. This study aimed to examine the roles of behavioral symptoms and vision loss on physical function recovery over one year among those with dementia after hip fracture by conducting a secondary analysis of 2,824 long-stay residents with dementia and behavioral symptoms from the Nursing Home Minimum Data Set 3.0 from 2017-2019. Longitudinal parallel process modeling was used to examine the longitudinal relationships among the trajectories of cognition, behavioral symptoms, vision loss, and physical function. Results showed that the initial status of cognitive impairment was associated with the initial status (β=-0.31, p < 0.0001) and rate of change (β=-0.26, P < 0.0001) of physical function. Behavioral symptoms partially mediated the longitudinal relationship between the initial status of cognitive impairment and the initial status (β=-0.01, p = 0.002) and rate of change (β=-0.01, P < 0.001) of physical function. Vision loss did not moderate such longitudinal relationships. The findings add new knowledge of how dementia and vision loss might longitudinally affect physical resilience among persons with dementia after a hip fracture.

